# Salmagundi

**Published:** 1886-07

**Authors:** 


### SALMAGUNDI.

EDITED BY E. G. BETTY, D.D.S.

DR. C. W. SPALDING, of St. Louis, is interested in some New Mexico mines.

A COUNTER irritant—the lady who prices everything and buys nothing.—*Clipping*.

CAMBRIDGE UNIVERSITY honored itself last month by conferring honorary degrees upon Dr. Oliver Wendell Holmes.

THE "Archives of Dentistry" beats the defunct "Dental Jairus" all hollow in the matter of editors, having no less than twenty-six. A few more would make one for each tooth in the head.

COL. R. M. HOE, the inventor of the double cylinder and the Web perfecting press, capable of printing 25,000 copies per hour, both sides, died in Florence, Italy, June 7th, aged 74 years.

Dr. J. G. CAMERON, of Cincinnati, mourns the loss of his son, W. Hart, who met his death in a railroad accident last month. Hart leaves a widow who has the sympathy of hosts of friends in her bereavement.

SIX women graduated from the Pennsylvania College of Dental Surgery at its Thirteeth Commencement. Of these, three hail from Germany, one from the West Indies, one from New York, and the last from Pennsylvania.

AVOIDING A SACRED SUBJECT.—A little girl, aged four, was sitting with a doll in her lap and a basin of water by her side, "What are you going to do with the dolly?" asked her mother.

" Christen her," replied the child. " Oh! you must not play at christening," returned the mother, " it is a sacred subject." " Then I'll vaccinate her, mamma ; that is not a sacred subject, is it ? "—*Vox Populi, Lowell, Mass.*

---

The bicycle is a means of exercise that combines both pleasure and the necessary muscular activity the dentist does not obtain in the practice of his profession. Dr. Callahan, of Hillsboro, is an expert, and heartily recommends the " machines" to all his dyspeptic friends, as a sure cure for all their ills.

---

We do not like to keep reminding our readers that they ought to contribute something now and then to *Salmagundi* in exchange for what they get so regularly each month, for fear they might blush too much upon seeing their names attached to an article in print. Most fellows like to see their patronymics blazoned forth in good honest type, but " Sal's" friends, you know, are too modest by half. We'd like to see the other half.

---

Dr. C. T. Stockwell, in a paper read before the Vermont State Dental Society, details a method of restoring teeth that have been broken down by " white decay," with a combination of amalgam and gold. He makes the statement (on the strength of some experiments of Dr. Miller's) that the electrical currents generated by such a conjunction of different metals makes " the micro-organisms that produce fermentation and putrefaction between and about the teeth," " simply " boycott" such teeth and spaces altogether, to the advantage, however, of the teeth themselves, as is indicated by results whenever this combination is used."

It is to be hoped so.

---

Cassell & Co., of No. 739 and 741 Broadway, New York, are issuing what they call "Cassell's National Library," edited by Prof. Henry Morley. The library is a series of volumes issued weekly, each one containing about 200 pp., small 16mo., on good paper and excellent type, that will not weary the eye, and at the extremely low price of ten cents per volume. The subjects embraced are legion, are drawn from strictly classical literature of all ages and climes, and so cannot fail to meet the tastes and inclinations of all classes of readers. We know of nothing at once so cheap and valuable, and can heartily recommend the series to the readers of the REGISTER when they wish to give their minds a rest and at the same time lay by a store of delightful information that will be a source of pleasure, and a fund to draw upon when filling a tooth for a literary patient. Try a volume or two, the investment will return an hundred fold.

---

THE composition of water in its historical aspect is a very curious and interesting study. Several claimants to the honor of its discovery only served to confuse the matter and lend it additional interest. When we were in partnership with Dr. Watt, in 1876, the subject was canvassed somewhat between us, upon the finding (in an obscure corner where it had become lost) of a large silver medal given to Jas. Watt, one of the discoverers, by the Royal Society. Dr. Watt at the time made the curious statement that the Jas. Watt who received the medal was not *the* Jas. Watt who made the discovery, but a cousin, both of whom were members of the family from which Dr. Watt is descended. We promised to write up the history of the discovery and did so, the sketch appearing in the December number of the REGISTER for 1879, Dr. Watt at the same time promising to make public the circumstances of the awarding of the medal to the wrong man. We hope that if the Doctor's eye falls upon this, it will stimulate him to redeem that promise in one of the " specials' 'he knows so well how to make interesting.

